# Early-career trajectories of young workers in the U.S. in the context of the 2008–09 recession: The effect of labor market entry timing

**DOI:** 10.1371/journal.pone.0214234

**Published:** 2019-03-26

**Authors:** Serge Atherwood, Corey S. Sparks

**Affiliations:** Department of Demography, University of Texas at San Antonio, San Antonio, Texas, United States of America; Universidade Federal de Minas Gerais, BRAZIL

## Abstract

**Objective:**

The goal of this study was to analyze differences in the employment and wage trajectories of college-educated young workers in the United States, as distinguished by the timing of their entry into the labor market relative to the onset of the 2008–09 recession.

**Methods and findings:**

Using annual American Community Survey microdata, we analyzed the first six years of employment and wage outcomes for cohorts of young workers on traditional-student pathways entering the market (1) in 2006, shortly before recession onset; (2) in 2009, during the recession; and (3) in 2012, three years after the recession officially ended. We found evidence for negative effects on outcomes and outcome trajectories differentiated by the recession’s proximity to workers’ labor market entry, including lower wages for the cohort entering in 2009. However, recession effects tended to be smaller for workers at the high end of the education gradient or with no direct exposure to the recession and were outweighed by gendered labor outcome disparities. We also observed a possibly enduring, recession-induced rise in the number of idle young males and the proportion of male and female high school graduates enrolled in college and not working.

**Conclusions:**

Cohort differences in labor outcomes show that the disadvantages of entering the labor market during an economic downturn appear lasting. However, the subordinate role of timing effects in sorting young workers’ employment and wage rates, when compared to the stark stratification of employment and wage outcomes by education or sex, is a useful reminder that these latter social structures remain key determinants of labor outcomes.

## Introduction

Each year in the United States, millions of young adults earn postsecondary academic credentials [1], motivated by the belief that the path to career success starts in higher education [2–3]. While these credentials are an important signaling device in the labor market [4–5], a successful start to young workers’ careers can also be influenced by factors beyond individual control. An important example is the business cycle. Recessions increase job competition and generate fewer opportunities to begin careers on a strong footing [6–9], and increase the risk of unemployment and underemployment [10–12] at a time when young adults are vulnerable to labor market setbacks as they try to establish independent lives [13].

Compared to previous downturns in the U.S., the “Great Recession” that officially lasted from December 2007 to June 2009 and caused approximately 8 million jobs to be lost [14] is notable for its exceptionally adverse impact on the labor market. Unemployment rates in the U.S. surged more sharply during the recession, and receded more slowly after it, than at any other time since World War II [15–18]. The pain was felt widely among young adults; for example, employment among young adults born in 1982 or 1983 with only a high school diploma fell 10.4 percentage points between December 2007 and June 2009 to 78.1% [12]. In October 2009, when the U.S. unemployment rate peaked at 10.0%, unemployment among recently-graduated bachelor’s degree holders aged 20 to 29 reached 17.6%, or nearly double the rate from two years earlier [19].

Such statistics are informative of the weakness of the labor market as experienced at the time by young adults, but less is known about how they fared over the longer term [20]. Even though young adults affected by the 2008–09 recession are still early in their careers and their lifetime earnings remain an open question, this knowledge gap deserves attention because evidence shows that entering the labor market in a downturn can hinder wage growth and cumulative earnings for years or even decades [7, 21–24]. If early career conditions offer clues to future labor outcomes, what might the conspicuously difficult conditions of the Great Recession suggest about the long-term economic performance of young workers who encountered the recession head-on? And, on the timing of labor market entry, how much difference might a few years have made in the way the recession shaped the early trajectory of a career? A decade removed from the 2008–09 recession, enough time has passed to allow us to begin considering these two questions.

In this study, we analyzed two labor outcomes—annual employment rates and annual wages—over a six-year period for three cohorts of young adults graduating in the late 2000s and early 2010s, stratified by sex and grouped by education attainment level. Cohorts were drawn from a pseudo-panel generated from American Community Survey (ACS) microdata. To contextualize variability in outcomes relative to the timing of the economic downturn, we staggered the timing of each cohort’s recession exposure to simulate labor market entry before, during, and after the recession, respectively. Variability was interpreted through the education—labor outcome gradient, which describes how higher levels of education correspond to higher annual wages and better odds of employment and is often referred to as the *return to schooling* [25–26]. The notion of a gradient is one of the key developments in econometrics from the last half-century, with roots in the human capital models of Becker [27] and Mincer [28] and the market signaling model of Spence [29]. More than just establishing the positive correlation between amount of schooling and wage earnings [30], it explains how the investment in time and money in higher education is rationalized by the anticipated return on that investment in the labor market [25, 31]. With higher education having become a normative part of the transition to adulthood [32–33], examining early-career labor outcomes on an education gradient is doubtless relevant—especially if those outcomes respond dynamically to the timing of recession exposure. Some proportion of young adults will be affected by each future recession that comes to pass, but how different might the effect be if it is a peripheral, rather than direct, encounter?

This study helps to fill the gap regarding how young adults’ labor outcomes and outcome trajectories were affected by the 2008–09 recession [20]. Given the construction of our cohorts (explained in detail below), our findings are generalizable primarily to young adults who completed their formal educations on a traditional-student timeframe. Still, the study contributes a new way of considering the variability of early-career trajectories launched at different points relative to a major exogenous shock and offers insights into the shape and resiliency of the education gradient for a recent generation of young workers. It also contributes clues to several potentially lasting changes in the proportions and behaviors of working, non-working, and college-going young adults after 2008.

On the basis of a long literature asserting the advantages of higher education in the labor market (e.g., [34–36]), we expected no recession-induced changes to the overall shape or ordinality of the gradient; regardless of entry timing, individuals with higher education credentials were better protected. We further conjectured that the recession influenced outcomes in cohort-specific ways, reflecting whether workers entered the market when the economy was strong, weak, or recovering. For young adults entering in 2006, during the mid-2000s economic boom, we expected job displacement in all education groups (but particularly among workers with the lowest degrees) and stalled wage growth after 2008. Considering the severity of the 2008–09 recession and the weakness of the subsequent recovery, we expected employment and wage growth to be especially sluggish for young workers entering in 2009, resulting in worse employment and wage outcomes, and more compression within the gradient, than either other cohort in any observation year. For young adults entering in 2012, when unemployment among recent college graduates had begun to decline [37], we expected a middle-of-the-road performance marked by entry into a still-weak labor market that was slowly returning to a normative macroeconomic state.

Our results supported many of these expectations, but not always in a cohort-specific way. Evidence for the gradient and its protective effect were strong, as workers with higher levels of education were generally less harmed by the recession and more advantaged during the recovery. But groups in the 2006 cohort did not have universally superior longitudinal outcomes despite the favorable conditions of their labor market entry, nor did groups in the 2009 cohort have universally worse outcomes despite their uniquely disadvantageous start. Additionally, recession effects at the cohort level were as often subverted by structural disparities in sex as by disparities in education attainment, suggesting that the education—labor outcome gradient was not the only phenomenon that can withstand even the deepest recessions.

## Materials and methods

### Data

The data used in this study come from the American Community Survey (ACS) public-use microdata series for each year between 2006 and 2017 contained in the IPUMS-USA database at the University of Minnesota Population Center [38]. The ACS is a demographic, housing, and workforce survey conducted by the U.S. Census Bureau on a national random sample of the U.S. population. While data from the U.S. Department of Labor’s Current Population Survey (CPS) is often used to study labor markets, use of ACS data is not without precedent (see, for example, [39–41]). One consideration when comparing ACS and CPS on labor indicators is the fact that ACS is a far larger survey: approximately three million households are sampled each year by ACS, compared to 100,000 by CPS [42]. The larger sample size of the ACS allows for greater reliability when analyzing groups within the sample. This was an important concern, given that forming our analytic sample ultimately excluded 79.2% of observations in an original IPUMS-USA data set of 9.4 million records. Slight differences between ACS and CPS exist in the way survey universes are defined, individuals in households are deemed eligible for interview, employment status questions are worded, and income is reported [43–44], but income estimates are highly comparable between the two surveys [43] and employment estimates have increasingly converged after 2007 [44]. Both are nationally representative samples as well.

Our analysis was restricted to noninstitutionalized civilians with a known birthplace, aged 18 to 34 years, and reporting having completed at least a high school diploma or equivalent at the time of survey. These criteria excluded individuals in the Armed Forces and individuals who did not reside in a Census-defined household or noninstitutional group quarters. Also excluded were individuals who had not yet graduated from high school. A small fraction of the ACS sample was further excluded after restricting race/ethnicity categories to the four largest groups: non-Hispanic white, non-Hispanic black, Hispanic, and Asian (with the latter category pooling respondents reporting as Chinese, Japanese, or Other Asian or Pacific Islander). To maintain consistency in our definition of employed persons as wage or salary workers with nonzero earnings, we excluded unpaid family workers and the self-employed. In the interest of complete records, we dropped 6,042 females (representing 0.2% of the total female sample before cohort selection) after cross-tabulation revealed missing information about their fertility status in the previous year.

#### Variables

Our outcomes of interest were current employment and annual wage, where employment and wage only applied to individuals who worked for an employer for pay. We coded employment at the individual level as a dummy variable, where being employed = 1. Mean annual employment rate was the proportion of all members in a cohort group who were employed full-time or part-time, whether they were also enrolled in college. Median annual wages were calculated for each cohort from the groupwise aggregation of annual wages reported individually for the previous 12 months at time of survey, adjusted for inflation to midyear (July) 2017 dollars using Consumer Price Index multipliers from the U.S. Bureau of Labor Statistics [45]. Extremely high-earning individuals were preemptively top-coded at the 99.5^th^ percentile by year by state in the IPUMS-USA data set [38], but to further reduce potential skewing we Winsorized the right tail of the wage distribution to the 98^th^ percentile of incomes reported by graduate degree-holders in the full analytic sample.

Our regressor of primary interest—level of education attainment—was a factor variable operationalized at four levels: high school diploma (HSD) or General Equivalency Diploma (GED), associate (2-year) degree, bachelor’s (4-year) degree, and graduate (master’s, doctorate, or professional) degree. HSD/GED was the reference group. Individuals who reported completing some college but no degree were coded as high school graduates.

We supplemented education attainment with eight independent variables as controls, of which five were dummy-coded: college enrollment (enrolled at time of survey = 1); birthplace (outside the U.S. = 1); having given birth in the previous year (yes = 1); part-time employment (less than 35 hours per week = 1); and employed at a job with a Siegel occupational prestige score of 40.6 or higher (yes = 1), where the score represented the median value for the full analytic sample. For each binary indicator, individuals for whom the indicator did not apply were coded as 0; e.g., all males were coded as 0 for the recent-birth variable, non-employed persons were coded as 0 for part-time employment, etc.

Remaining variables were race/ethnicity, observation year, and years of experience. Race/ethnicity was a factor variable with four levels: non-Hispanic white (reference group), non-Hispanic black, Hispanic, and Asian. Observation year was a six-level ordinal factor variable with levels corresponding to ACS survey years. The survey year in which an individual was observed for the first time was coded as 1 (reference level) and each subsequent year was a unit increase. This coding allowed us to measure elapsed time and capture period effects, although each cohort entering observation in a different survey year meant that the period effect of a given survey year was assigned to a different observation year and required cross-referencing prior to interpretation. Finally, on the assumption that individuals entered observation with no work experience (explained in the next subsection), we captured the effect of cumulative work experience by cloning the observation year variable as a continuous variable and subtracting 1 from each record to start the count at 0 years of experience.

#### Cohort assignment and attrition

Young adults are increasingly disposed to pathways that combine, alternate, or delay the roles of school and work and prolong their transition to adulthood [46–47]. This can make it challenging to know when a young adult has ended their educational career for good and/or entered the labor market in a “career” frame of mind and it has implications for how labor market entry and cohort membership should be defined. These concerns are particularly germane in recessionary contexts because individuals may intentionally delay their entry into the labor market in order to avoid or reduce their exposure to a recession.

One approach to addressing the endogeneity of timing entry is to focus the analysis on cohorts solely composed of individuals selected on criteria that reasonably preclude the possibility of delayed timing. This can be done by identifying cohorts in the mold of the traditional student, for whom the roles of student and worker are sequential and the demarcation between schooling and labor market entry is clear [48]. The term *traditional* may be something of a misnomer in light of how heterogeneous the transition to adulthood has become for many young adults today [49], but it reflects an (idealized) progression of milestones for students taking a linear “school before career” path to adulthood. Importantly, it is a progression that can be tied to age, which can be used as a cohort eligibility criterion. If the average age at high school completion can be assumed as 18 or 19 years, a traditional postsecondary pathway would have a high school graduate matriculate within a year or two and complete an undergraduate credential approximately two years later (for an associate degree) at around age 21 or 22, or four years later (for a bachelor’s degree) at age 23 or 24. If a recently-graduated four-year college graduate continued on for a graduate degree, at least two or three more years would pass before that student concluded their education for good and transitioned to the labor market.

By this logic, we can create synthetic cohorts from our analytic sample to follow through time in approximately the same manner as a panel study using longitudinal data [50–51]. This “pseudo-panel” approach is a familiar method in labor economic research for working with cross-sectional data (e.g., [52–55]). We can also begin observing each cohort in a different year to expose it to the recession at a different time. Hence, we created a “pre-recession” cohort (C1) that entered the labor market in 2006, a “recession” cohort (C2) that entered in 2009, and a “post-recession” cohort (C3) that entered in 2012. Although only a few years separate one cohort’s entry from the next, each entry year represents a different macroeconomic environment: 2006 was the peak of the mid-2000s boom, 2009 was the nadir of the recession, and 2012 was when the recovery began to gather strength. Next, we sorted eligible members of our analytic sample into the three synthetic cohorts according to the birth year that matched the expected age for each education attainment level for the year when each cohort was first observed (Table 1). Because the first year of observation for each cohort was spaced three years apart, we defined expected ages as three-year spans: high school diploma or GED at ages 18 to 20, two-year associate degree at ages 21 to 23, four-year baccalaureate at ages 24 to 26, and graduate degree at ages 27 to 29. While narrow, these expected age ranges are consistent with an on-time high school graduation at age 18 or 19 and with studies predicating that most college-goers who finish undergraduate degrees do so by their mid-20s; e.g., [8–9, 39]. Importantly, these age ranges—upon translation into birth year ranges—preserved mutual exclusivity in each education level within and between cohorts, which was essential for producing consistent cohort-level estimates in our models [50–51]. Of the 5,862,332 records in the analytic sample, one-third of them (1,953,876 records) matched on birth year and education attainment level and were assigned to a cohort.

**Table 1:** Expected birth year ranges by education level and cohort.

*Highest education level*	Cohort 1 “Pre-recession” (entered 2006)	Cohort 2 “Recession” (entered 2009)	Cohort 3 “Post-recession” (entered 2012)
HSD/GED	b. 1986–88	1989–91	1992–94
2-year degree (Associate)	b. 1983–85	1986–88	1989–91
4-year degree (Bachelor’s)	b. 1980–82	1983–85	1986–88
Graduate degree	b. 1977–79	1980–82	1983–85

Note: Expected birth year ranges assume a traditional-student pathway, whereby college enrollment follows high school graduation without a lengthy delay and continues without interruption until the desired academic credential is achieved.

Individuals assigned to a cohort were also sorted into four mutually exclusive categories of work/nonwork status: worker only; student/worker; student only; and not in employment, education, or training (NEET). Sorting was based on the cross-tabulation of ACS variables indicating labor force participation and college enrollment status. Individuals reporting unemployment or no labor force participation at time of survey were coded as NEET unless they also reported being enrolled in college, whereupon they were coded as student only. No distinction was made in the status variable between full-time and part-time employment among individuals coded as worker only or student/worker.

To generate a more pragmatic count of workers and nonworkers, and in the interest of sample consistency, we cross-referenced our status variable with individuals’ wage earnings. If, in a given survey year, an unemployed person reported wage earnings, we coded that person as employed on the assumption that those earnings indicated that person was employed at some point in the previous 12 months but not specifically at the time of survey. Worker only and student/worker were the only categories corresponding to being employed in our employment outcome variable.

Table 2 summarizes cohort composition by education attainment, work/nonwork status, college enrollment status, employment status, and sex ratio (M:F) for the first year of observation. We use the first year because an essential presumption we make about our pseudo-panel is that observed individuals completed the credential appropriate to their age as traditional students and thus had no work experience when we observed them for the first time in 2006, 2009, or 2012. In that regard, everyone in a cohort entered observation on the same footing; i.e., as labor market novices [7], and we can argue that the first observation of employment, whether in year 1 or later, was their first entry into the labor market following the completion of their schooling [56]. If we further assume that the attributes that selected individuals into observation were fixed, we could treat each cohort as a form of stationary population for which there would be no change in size, age distribution, or composition from one year to the next [57]. (For simplicity, we ignore population change from causes such as migration or death and the minor variability that may occur from re-estimating the sample size each year).

**Table 2:** Cohort composition in first observation year (selected variables).

	Cohort 1 (entered 2006)		Cohort 2 (entered 2009)		Cohort 3 (entered 2012)	
Variable	*n*	Sex ratio	*n*	Sex ratio	*n*	Sex ratio
*Highest education level*						
HSD/GED	84,476	0.954	90,671	0.959	90,961	0.983
% of cohort	68.3		67.0		65.9	
2-year degree	7,685	0.814	7,851	0.825	8,489	0.824
% of cohort	6.2		5.8		6.2	
4-year degree	24,146	0.771	27,411	0.795	28,183	0.821
% of cohort	19.5		20.2		20.4	
Graduate degree	7,394	0.698	9,497	0.636	10,292	0.643
% of cohort	6.0		7.0		7.5	
*Work/nonwork status*						
Worker only	50,743	0.996	52,072	0.973	51,703	1.002
% of cohort	41.0		38.4		37.5	
Student/worker	52,333	0.806	53,510	0.785	50,714	0.800
% of cohort	42.3		39.5		36.8	
Student only	12,918	0.921	18,699	0.945	23,298	0.927
% of cohort	10.4		13.8		16.9	
NEET	7,707	0.788	11,150	0.961	12,209	0.978
% of cohort	6.2		8.2		8.9	
*College enrollment*						
Enrolled	65,251	0.828	72,209	0.824	74,012	0.838
% of cohort	52.7		53.3		53.7	
Not enrolled	58,450	0.966	63,221	0.971	63,912	0.997
% of cohort	47.3		46.7		46.3	
*Employment status*						
Employed	103,076	0.895	105,581	0.873	102,418	0.897
% of cohort	83.3		78.0		74.3	
Not employed	20,625	0.869	29,849	0.951	35,506	0.944
% of cohort	16.7		22.0		25.7	
**Cohort size (sex ratio)**	**123,701**	**0.890**	**135,430**	**0.825**	**137,925**	**0.909**

Source: IPUMS-USA (ACS PUMS 1-year samples, person-weighted).

Note: All counts sum to cohort size and all percentages sum to 100 within each variable. Sex ratio is the proportion of males to females.

But this assumption is problematic. While birth year is certainly a fixed attribute for individuals, education attainment is not. It is implausible that no cohort members acquired higher credentials during their six years of observation. Certainly, the proclivity for higher education among young adults means we should expect a substantial number of high school graduates observed at market entry to earn postsecondary degrees within the next five years. But any person earning a higher credential before being right-censored becomes ineligible for further observation in their original cohort because the age at which they earned the higher degree violates the traditional-student assumption required to originally observe them in that cohort. We must expect cohort sizes to change over time as individuals are lost to observation.

Given the mutually exclusive birth-year structure of our pseudo-panel, some of the attrition in one cohort will be captured in a later cohort. However, because each annual cross-section of cohort members is drawn from a different population sample, it is impossible to follow individuals through time and know how much attrition is being recaptured. Fortunately, if the groupwise attrition pattern is reasonably consistent across cohorts from one observation year to the next, we may assume there is no substantive change in how groups are constituted over observation time. This is because in a pseudo-panel the basis for group membership remains fixed at the group level, making the *group* the unit of analysis instead of the individual [58]. Even if a group (education attainment level) loses individual members to attrition, remaining members still share the same birth year range and education requirements.

**Fig 1:**
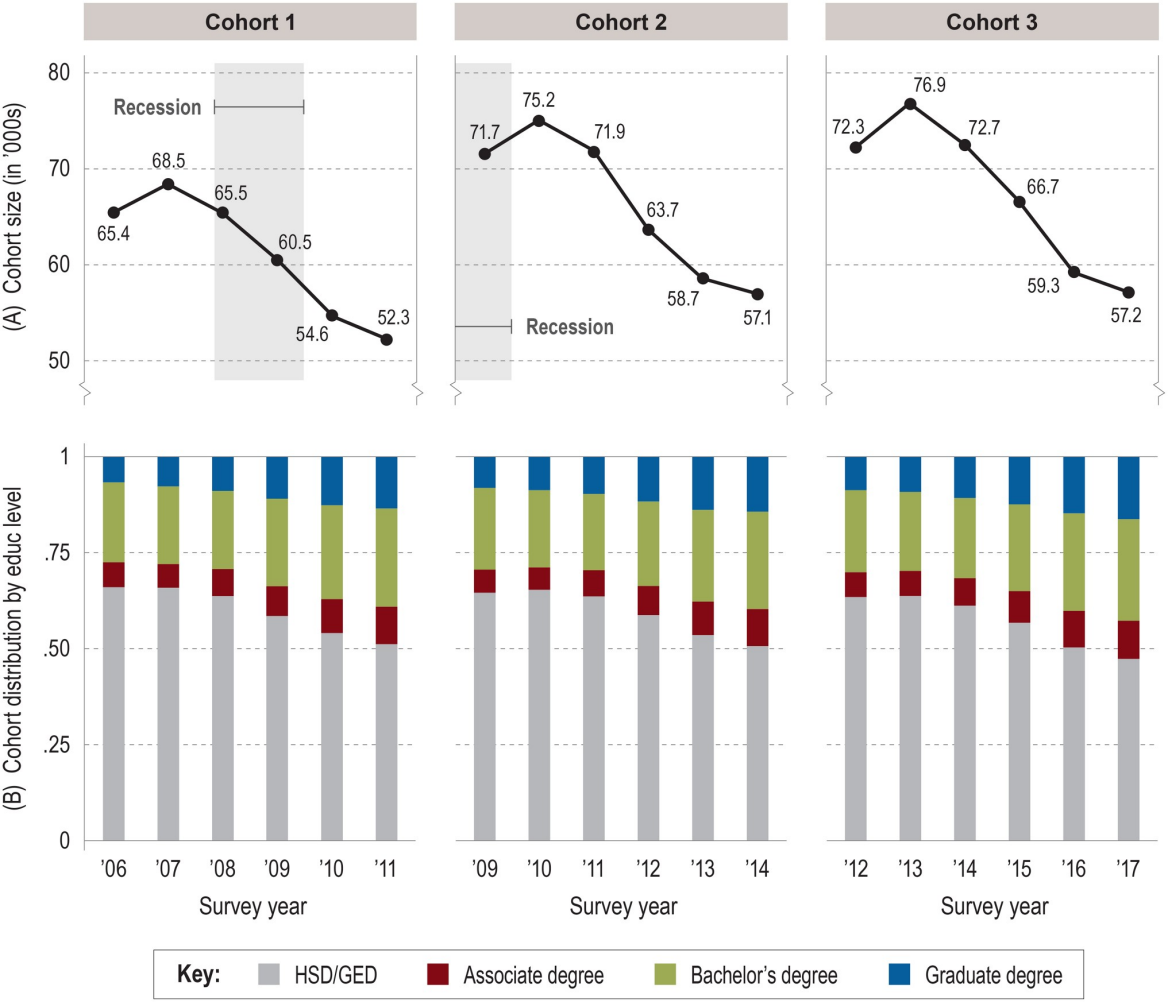
Size and distribution of female cohorts by education level and survey year. (A) Person-weighted total size of cohort in survey year. (B) Distribution of cohort by education level as proportions of cohort population. Source: IPUMS-USA (ACS PUMS 1-year samples, person-weighted).

**Fig 2:**
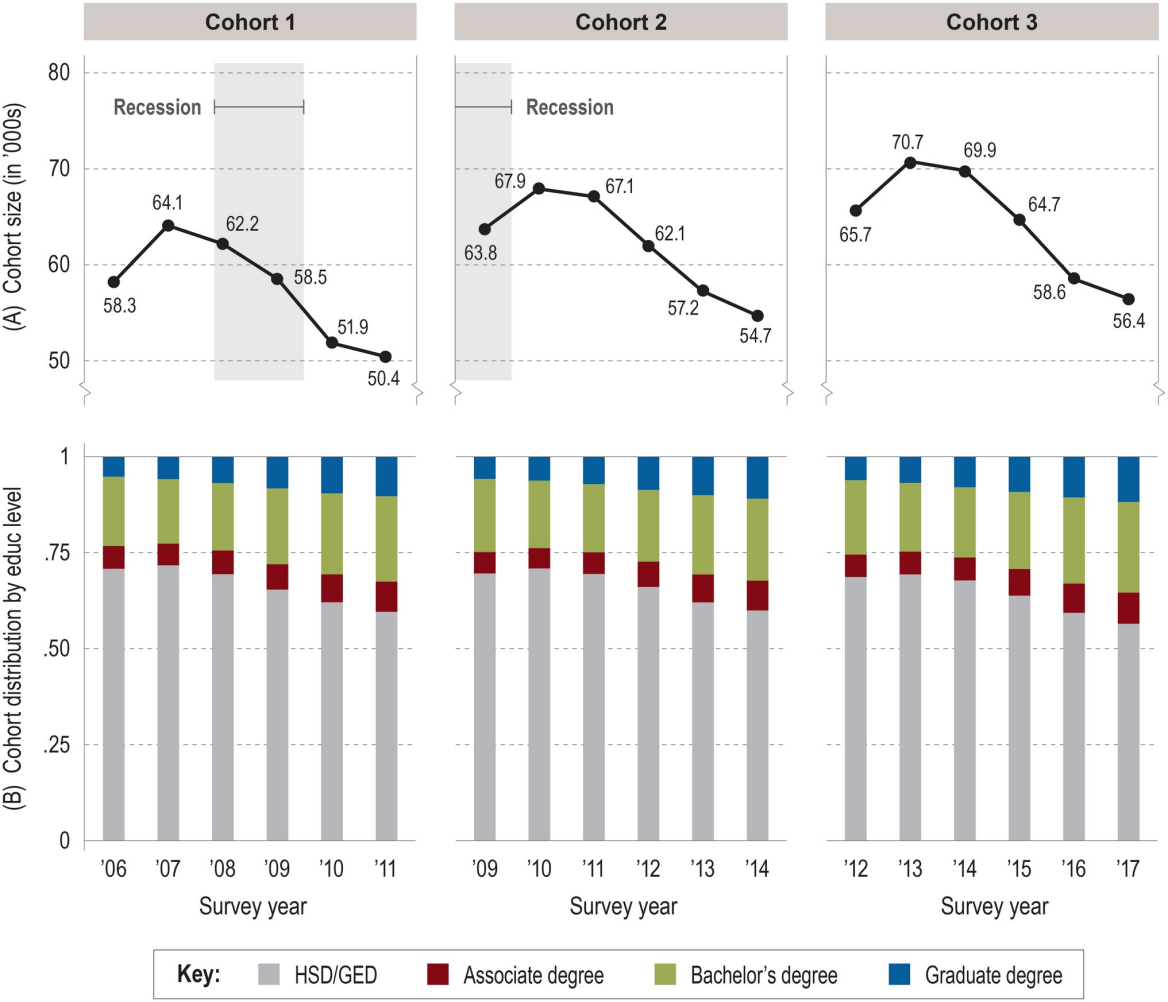
Size and distribution of male cohorts by education level and survey year. (A) Person-weighted total size of cohort in survey year. (B) Distribution of cohort by education level as proportions of cohort population. Source: IPUMS-USA (ACS PUMS 1-year samples, person-weighted).

Figs 1 and 2 show the size of the cohort population at each level of education by survey year for females and males, respectively. By the end of observation, the (weighted) overall size of female cohorts (Fig 1A) was approximately one-quarter smaller, on average, than their peak size in year 2, while male cohorts (Fig 2A) were about one-fifth smaller. Most of the attrition was in the HSD/GED group; as the cohort population declined over time, so too did the proportion of high school graduates. Female cohorts showed more attrition than males; their proportion of HSD/GED fell from 65% when first observed to 51% or less at end of observation (Fig 1B), while among males this proportion fell from around 70% of the full cohort to 60% or less (Fig 2B).

Regardless of cohort or sex, the longitudinal HSD/GED attrition pattern necessarily dovetailed with growth in the proportions of young adults earning higher degrees. With their larger cohort sizes, females consistently outnumbered males in postsecondary degrees earned each year, but the disparity in higher education attainment is especially illustrated by the consistently greater *proportion* of females with higher degrees compared to males in any given cohort or year. Because we analyzed our cohorts separately by sex, these disparities did not represent an attrition problem between cohorts for the purposes of our study. Similarly, the slight yet highly comparable increase with each later cohort (male or female) in the proportions of individuals with postsecondary degrees during the first year of observation allowed us to conclude that our cohorts were appropriately consistent through time.

### Empirical strategy

All statistical analyses were conducted in the R statistical programming language [59] with the aid of specific libraries identified below. For greater representativeness of the U.S. young adult population, we weighted our data using person-weights provided by IPUMS-USA [38]. On the basis of distinct (although narrowing) gender differences in the transition to adulthood and early career (e.g., [60–63]), we analyzed labor outcomes for males and females separately.

We began by describing mean annual employment rates and median annual inflation-adjusted wage trajectories for each cohort by education attainment level, using the “survey” library [64]. We tested groupwise differences in trajectories within and across cohorts using two-way ANOVA and Tukey multiple pairwise tests. To assist in interpreting trajectories, we also described the distribution of work/nonwork status levels by cohort and observation year.

We then turned to estimating the effect of education attainment on mean employment and wage outcomes. It is well-established that education attainment is positively correlated with both employment (e.g., [65–66]) and wage (e.g., [67–68]) and it is obvious that wages are earned by the employed. These facts lie at the heart of a classic endogeneity problem involving self-selection: employed individuals tend to earn higher wages if they have higher levels of education, but it is precisely those individuals with higher levels of education who tend to be employed in the first place. This self-selectivity into employment (and higher wages) makes it likely that the education gradient of employed individuals will not be representative [66, 69–70], but the cause behind such selectivity (often described along the lines of natural or innate ability) is an unobserved variable positively correlated with education attainment and on-the-job performance: individuals with greater ability are often predisposed to using their ability to obtain higher levels of education *and* earn higher wages regardless of their education level [26, 71].

This situation gives rise to our endogeneity problem. In a classically-structured ordinary least squares (OLS) model regressing wage on education, education is the predictor variable while ability—being unobserved—is an omitted variable whose effect on wage is captured in the error term [72]. If, in a sample of workers, those with greater ability have higher levels of education and higher wages, the estimated values for the predictor and error terms will both be larger. In other words, the predictor variable will be positively correlated with the error term, which will bias the estimator upward [26, 73–74]; we will assign more influence to education attainment than it actually has. This is a problem because we wish to estimate what the *unconditional* mean wage would be based on education attainment data from *all* individuals in our sample—including those (censored) individuals who did not work—not the mean wage directly conditioned on being employed and indirectly conditioned on education attainment.

To reduce this bias in the marginal effect on wage for different levels of education, we implemented a two-stage Heckman-type sample selection model. Specifically, we used the “sampleSelection” library [70] to generate a Tobit-2 (Heckit) model for jointly estimating a (first-stage) *selection equation* for the individual likelihood of being employed and a (second-stage) *outcome equation* for mean annual log wage. The selection equation is a probit model that predicts the likelihood of selection (employment) for each cohort member and identifies, in the form of the inverse Mills ratio (IMR), their individual selection hazard. These IMRs are used as the omitted regressor in the outcome (wage) equation—one that informs the equation on the estimated likelihood of each person in a sample to be employed [74–76].

In accordance with standard practice for ensuring the selection equation is adequately identified, we employed exclusion restrictions; i.e., variables (instruments) that correlate with self-selection into the non-censored group providing the data on the outcome of interest, but not with the outcome itself [70, 75]. We chose four regressors to predict selection into employment yet not directly influence wages: (1) having recently given birth, (2) place of birth (U.S. or non-U.S.), (3) college enrollment status, and (4) observation year. A recent birth is more likely to influence whether a person opts to temporarily withdraw from the labor force than their wage rate if they stayed employed [77–78]. Birthplace may be a more appropriate predictor of employment than wage given that a substantial share of U.S. immigration is employment-based [79] and wage discrimination based on national origin is unlawful [80]. College enrollment status may instrument for selectivity into employment since employment can compete with school for a person’s time and attention [81–82]. Indeed, many full-time students do not work while attending school [83]. We might also expect a joint effect from birthplace and college enrollment, given the propensity for many foreign-born young adults to study at U.S. postsecondary institutions [84]; an interaction term will allow us to capture it. Lastly, if we theorize that unemployment is a product of the macroeconomic climate (a period effect), observation year can be a proxy for the economic conditions of each survey year.

The exclusion restriction extends to the outcome equation as well—only inverted, since we want instruments that correlate with wage but not with likelihood of employment. We chose four variables for exclusion: (1) part-time employment, (2) occupational prestige, (3) years of experience, and (4) race/ethnicity. The merits of the first three variables are self-evident, although our measure of work experience assumes a traditional-student pathway for every observed individual. (We also included as a regressor the square of years of experience to account for nonlinear effects as experience accumulated over time.) Meanwhile, we assigned race/ethnicity as a regressor in the outcome equation on the basis of its established and persistent negative correlation with wage (e.g., [85–86]).

## Results

### Descriptive results: Mean annual employment rates

#### Females

**Fig 3:**
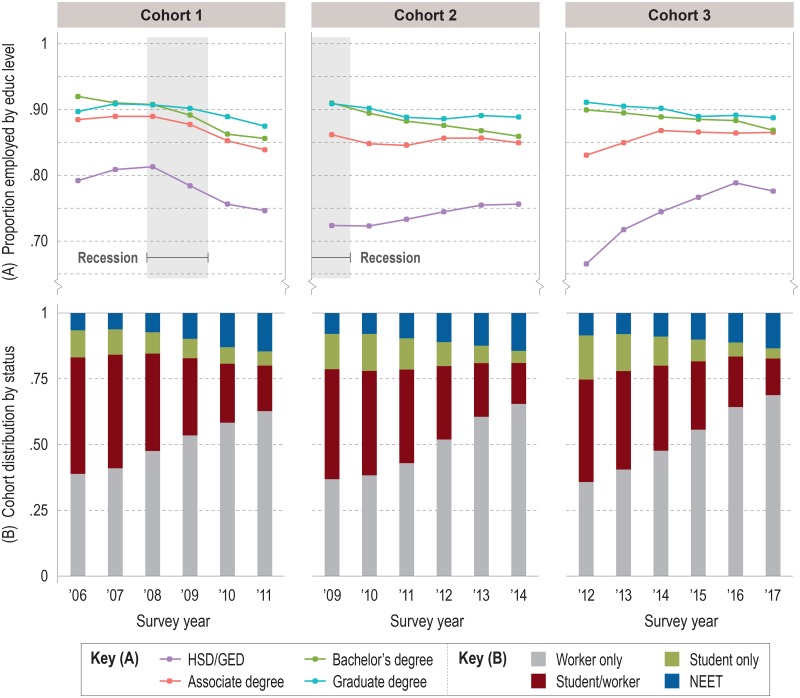
Employment rates and distribution of work/nonwork status for female cohorts. (A) Mean annual employment rates by cohort, stratified by education group. (B) Distribution of cohort by work/nonwork status as proportions of cohort population. Note: Employment represents part-time or full-time work for wages or salary. Self-employed individuals are excluded from the cohort population. Source: IPUMS-USA (ACS PUMS 1-year samples, person-weighted).

Fig 3A shows mean annual employment rate trajectories for the three female cohorts, where rates are equivalent to the proportion of the cohort population working full-time or part-time for wages or salary. Trajectories are stratified by level of education. These plots are paired with graphs (Fig 3B) describing the annual distribution of each cohort across four categories of work/nonwork status. Post-hoc ANOVA and Tukey tests showed trajectories were statistically significantly different between all cohort pairings except C2–C3 and all group pairings except bachelor’s degree—graduate degree.

Broadly speaking, the recession coincided with a decline in C1 female employment in year 4 (2009) that continued until the end of observation. All groups were affected to varying extents. High school graduates experienced a drop of almost 5 percentage points (ppts) between 2008 and 2009, the steepest loss among groups. But it was bachelor’s degree-holders who showed the greatest longitudinal decline. From 92% employment in 2006 to 85.5% employment in 2011, their groupwise mean rate was falling two years before the recession occurred. The recession’s immediate effect can be seen as well in the lower mean employment rate of C2 females entering the labor market in 2009 compared to C1 entrants in 2006 (especially among HSD/GED). Employment did not change substantially over the observation period except for bachelor’s degree-holders, who again showed steady annual rate declines and the largest cumulative drop between 2009 and 2014. From the same starting employment rate above 90% in 2009, bachelor’s and graduate degree trajectories diverged after the latter group stabilized in 2011 at around 88%.

Employment rates among C3 groups were perhaps the most varied: significant employment weakness in 2012 among HSD/GED and associate degree-holders was rapidly overcome in subsequent years, making them two of only three groups among female cohorts to end observation with a higher mean employment rate than they started with. Graduate degree employment followed the shallow longitudinal decline of their peers in C2, while bachelor’s degree employment fell in similar fashion, finally bucking the pattern of the previous two cohorts.

Relative to 2006, the fall in initial employment rates for HSD/GED and associate degree-holders in 2009 and 2012 is conspicuous. Fig 3B suggests where these potential wage-earners were during the recession or after it: in college or idled at home. The proportions of the student only group in C2 in 2009 and 2010 were 3.1 and 4.4 ppts greater than those for C1 in 2006 and 2007, respectively—representing groupwise growth of 30% in the first year and 46% growth in the second year. Comparing the groupwise proportions of C1 and C3 during the first year of observation is even more striking: at 16.7% of the cohort population, the C3 student only group was 62% larger than the C1 equivalent. A similar picture emerges with the proportion of NEET in each cohort, although the amount of yearly change was smaller and the recession effect was modest relative to the longitudinal growth trend in this group. Although C2 and C3 showed slightly larger initial proportions of NEET compared to C1, by year 6 all three cohorts had similar proportions of NEET (14.1%, on average). We note an interesting recession-linked relationship between the growth rates of worker only and NEET groups that resulted in each cohort with about the same NEET proportion at end of observation: in C1, the overall growth rate among NEETs was substantially greater than among workers only—not an unexpected outcome given the late timing of the recession for individuals entering in 2006. By contrast, the same two groups in C2 grew at about the same overall rate, while C3 NEETs grew at a slower rate than workers only.

#### Males

**Fig 4:**
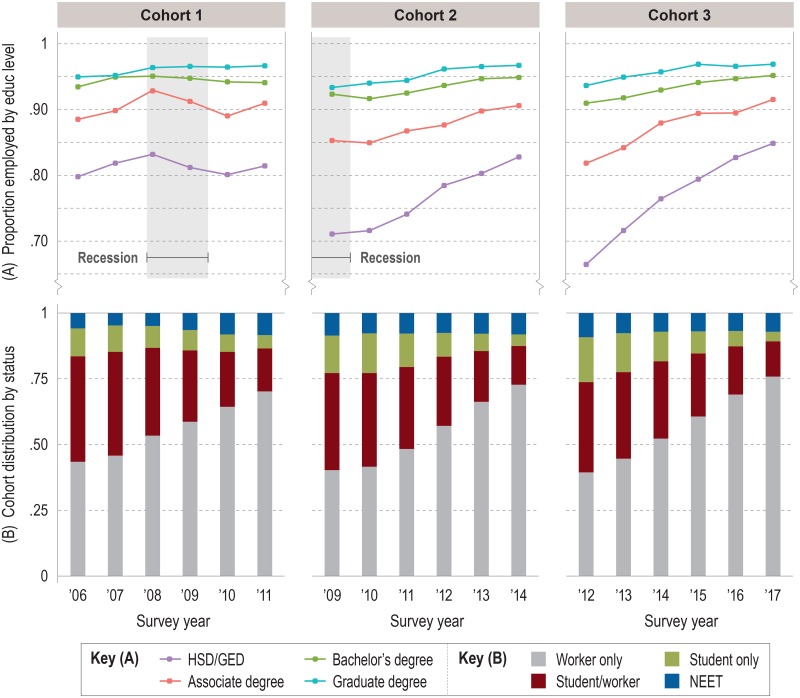
Employment rates and distribution of work/nonwork status for male cohorts. (A) Mean annual employment rates by cohort, stratified by education group. (B) Distribution of cohort by work/nonwork status as proportions of cohort population. Note: Employment represents part-time or full-time work for wages or salary. Self-employed individuals are excluded from the cohort population. Source: IPUMS-USA (ACS PUMS 1-year samples, person-weighted).

Male employment trajectories and cohort distributions are shown in Fig 4. In many ways, males experienced the recession as females did, but several differences stand out. For example, in 2008, employment decline among C1 males only occurred among individuals with high school diploma/GED or associate degree as their highest credential (Fig 4A). And while both groups lost 3 to 4 ppts in average annual employment rate by 2010, both returned to growth in 2011 (contrary to their female peers). The steady decline in employment rate seen for almost all female groups was reversed for all male groups except the two C1 groups just described.

As was the case with female cohorts, cumulative growth was strongest for associate degrees and high school graduates in C2 and C3. Males with bachelor’s degrees were consistently employed at nearly the same rate as males with graduate degrees, in another echo of female trends. However, males in the lower half of the education gradient experienced more growth than their female peers, and male groups with the highest degrees often showed incremental growth instead of incremental decline.

The female pattern of sharply lower employment at labor market entry for high school graduates entering during or after the recession (and to a lesser extent associate degree-holders) was also seen for males (Fig 4A), and for the same reasons: young adults delaying their entry into a weak labor market by enrolling in college, or young workers unable to find jobs and being idled at home. Somewhat surprisingly, given the lower likelihood of males to earn academic credentials, student only males in C2 and C3 comprised nearly the same proportions of their cohorts as their student only female counterparts in the first two years of observation (Fig 4B). Equally interesting is the recession-induced trend among C1 NEET males: in 2007 and 2008, they represented no more than 5.8% of the C1 cohort before climbing approximately 2.5 ppts by 2011 to 8.4%—where the proportion would remain, plus or minus a percentage point, for the remainder of their observation period and for the entirety of C2 and C3. This is perhaps the most prominent evidence so far for a long-term recession effect that affected males more negatively than females.

Regardless of cohort, males with bachelor’s or graduate degrees experienced the least amount of employment volatility of any group or sex, at least relative to the high-water marks set by C1 individuals during their first two or three years of observation, before the recession. C1 males at the two highest levels of education entered in 2006 at about 94–95% employment and largely remained there throughout observation. (This is in sharp contrast to females with the same high levels of education, all of whom entered with lower average annual employment rates of around 90–91% and were right-censored at lower rates than they started with.) But even among males in the upper half of the education gradient, those with graduate degrees managed to build distance from those with bachelor’s degrees. Whereas the recession caused employment in the latter group in C1 to stall and decline (however slightly), the former group held fast at about 96% employment for four straight years. And in 2010, graduate degree-holders in C2 avoided the minor bump in joblessness that befell bachelor’s degree-holders, resulting in a 2–3 ppt employment rate deficit that persisted in C2 for the next four years and was observed in all six years of C3.

### Descriptive results: Median annual wages

**Fig 5:**
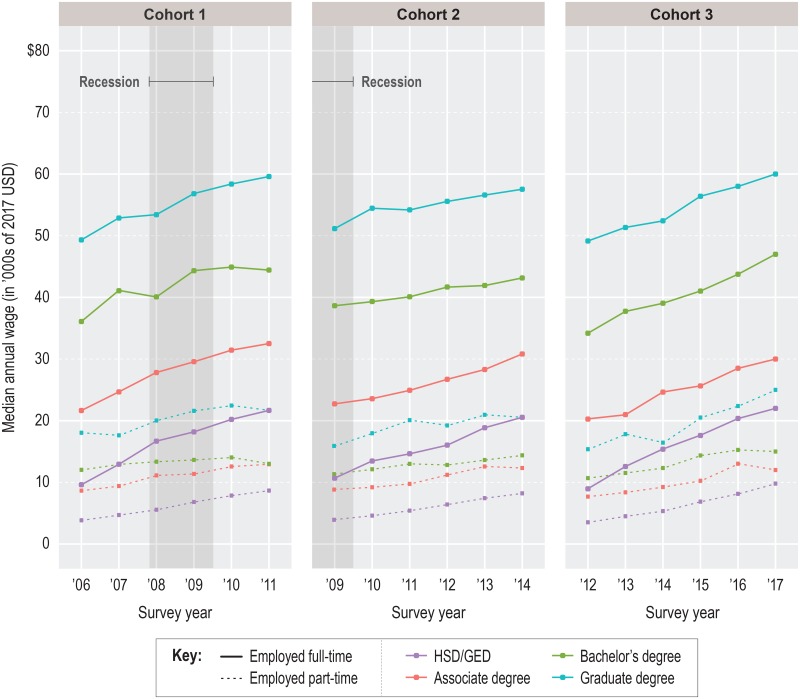
Female median annual wages by cohort and group. Note: Part-time employment = less than 35 hours per week; full-time employment = 35 or more hours per week. Self-employed individuals are excluded from the cohort population. Wages are Winsorized on the right tail at the 98^th^ percentile of graduate earners. Source: IPUMS-USA (ACS PUMS 1-year samples, person-weighted).

**Fig 6:**
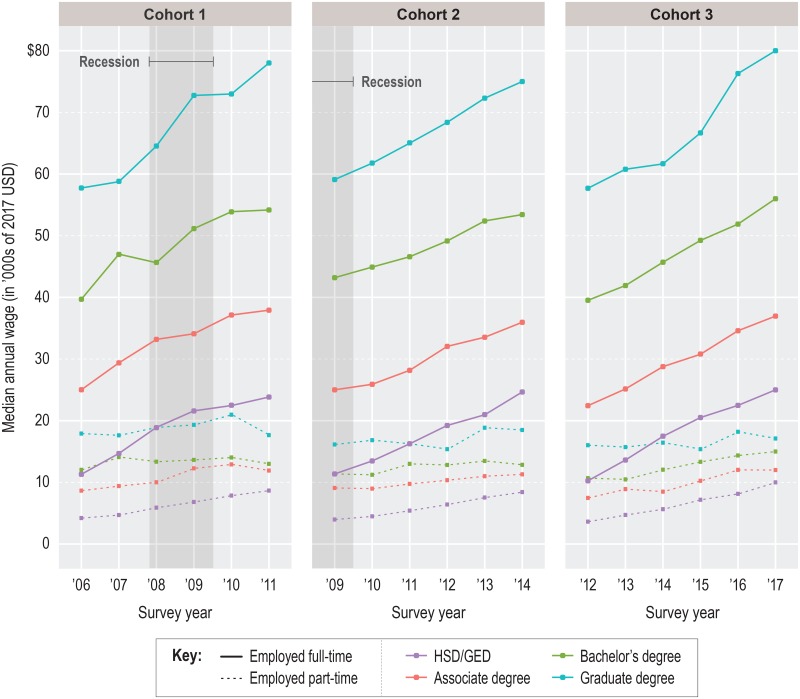
Male median annual wages by cohort and group. Note: Part-time employment = less than 35 hours per week; full-time employment = 35 or more hours per week. Self-employed individuals are excluded from the cohort population. Wages are Winsorized on the right tail at the 98^th^ percentile of graduate earners. Source: IPUMS-USA (ACS PUMS 1-year samples, person-weighted).

We show young workers’ wage trajectories in Fig 5 (females) and Fig 6 (males). Wage data were adjusted for inflation but otherwise unconditioned. Solid lines indicate the estimated groupwise median annual wage for full-time (FT) workers and dashed lines indicate the same for part-time (PT) workers.

In contrast with the sex-dependent divergence in employment trajectories, median annual wages grew over time for both males and females and fell along the same education attainment gradient, with higher credentials earning higher wages. With relatively few exceptions, wage growth occurred annually for FT and PT workers alike. Direct recession effects were largely absent; wages grew from 2008 to 2010 for C1 workers in the third and fourth years of their careers and, rather surprisingly, almost all C2 FT workers entered observation in 2009 with *higher* median wages than their C1 peers in 2006. (It must be noted, though, that C2 wages in year 6 were often lower than C1 or C3 wages in year 6, which fits with evidence for a recession-specific scarring effect on wage growth; e.g., [21–24].) Regardless of cohort or sex, median FT wages for bachelor’s degrees or higher were only a few thousand dollars greater than the pooled median for FT and PT workers [not shown]. This is attributable to the much smaller median wages for PT workers across education levels—often only one-quarter to one-third of FT amounts—and the large ratio of full-time workers to part-time workers (Table 2). The recession had minimal impact on this.

Despite the numerous ways wage trajectories were similar for males and females, they also differed by sex in three interrelated areas. First, female FT workers showed significant wage compression compared to males; from lowest to highest level of education, female median annual wages varied by only about $40,000 in any given year, while male wages varied by up to $55,000. Constrained by a narrower wage range, female workers experienced smaller wage premiums for each higher level of education. As an example, male graduate degrees earned an average of $14,500 more in FT median wage per year than male bachelor’s degrees, which themselves earned up to $17,415 more than male associate degrees, on average (Fig 6). For females with the same credentials, the dollar amount of each FT premium was up to one-third smaller.

Second, FT median annual wages at initial labor market entry were consistently higher for males, regardless of group. For all groups except graduate degree-holders, FT female workers pooled across cohorts showed a gender wage gap equal to about 12.3% of the FT female median wage. The gap was 16.7% at the graduate degree level. Among PT workers, most groupwise gaps were nonexistent or nearly so. However, this wage parity is overshadowed by the fact that female workers were more likely than male workers to be working PT [not shown]. Among young adults who were in the worker only category, the male PT:FT ratio was as much as 74% smaller than the female PT:FT ratio in a given observation year. (One direct effect of the recession was a reduction in the size of this gap at each level of education for C2 and C3 workers relative to C1 workers, caused by the greater proportion of C2 and C3 worker only males in all groups below graduate degree who worked PT when they first entered observation. But this convergence was short-lived, having mostly stalled by year 4).

Third, males tended to show greater median annual wage growth than females. This is particularly evident when contrasting the median wages of FT males and FT females in the two highest education groups. Median annual C1 wages for females with bachelor’s degrees averaged 4.5% growth relative to the previous year, compared to 6.7% for equivalent males. For C2 workers with bachelor’s degrees, the recession cut the annual growth rate for females by half (to an average of 2.2%) but only by one-third for males (to an average of 4.4%). A similar pattern of male advantage was observed among workers with graduate degrees.

The general consequence of these various gendered differences was a growing disparity in median wage earnings from the very start of observation, sometimes resulting in shockingly large wage gaps. A male with a bachelor’s degree entering the labor market in 2006, for example, earned a median wage of $39,696 compared to $36,087 for a female with a bachelor’s degree (all else equal). In 2011, the same male worker earned $54,175 (36.5% gain) while the same female worker earned $44,424 (23.1% gain). A male with a graduate degree started in 2006 with a median wage premium of about $18,044 relative to a male with a bachelor’s degree; by 2011, the premium had grown 46% to $23,837. For females in the same context, the premium grew only 15%, from $13,232 to $15,169. Similar widening gaps were seen in other education attainment groups and across cohorts, indicating a universal pattern. It is important to bear in mind that these wage disparities may have been influenced by uncaptured or unobserved conditions. Nonetheless, they clearly suggest that the education—labor outcome gradient was not gender-equal for young adults before, during, or after the recession.

### Selection model results

Tables 3–6 show results for the two stages of the Heckit model by cohort, stratified by sex. Coefficients from the first-stage probit equation estimate the latent individual likelihood of being employed in a given year and are presented for males and females in Tables 3 and 4, respectively. Coefficients from the OLS multiple regression equation estimate the size of the mean annual log wage and are shown for males and females in Tables 5 and 6, respectively. We begin, however, with a review of model fit. Below, we use the term *significant* to refer solely to statistical significance at the 95% confidence level, and *substantial* (or related terms) to describe effect size.

**Table 3:** Probit selection equation results for male likelihood of employment.

Variables	Cohort 1 (observed 2006–11)		Cohort 2 (observed 2009–14)		Cohort 3 (observed 2012–17)	
	*β*	SE	*β*	SE	*β*	SE
*Highest education level*						
Associate degree	0.398***	0.014	0.434***	0.012	0.416***	0.012
Bachelor’s degree	0.653***	0.010	0.720***	0.009	0.699***	0.009
Graduate degree	0.871***	0.017	0.928***	0.015	0.936***	0.015
*Observation year*						
Year 2	0.069***	0.010	0.000	0.009	0.122***	0.009
Year 3	0.110***	0.010	0.062***	0.009	0.242***	0.009
Year 4	0.010	0.010	0.167***	0.010	0.310***	0.009
Year 5	−0.051***	0.011	0.208***	0.010	0.365***	0.010
Year 6	−0.041**	0.011	0.249***	0.010	0.407***	0.011
*Birthplace*						
Outside the U.S.	−0.127***	0.013	−0.098***	0.013	−0.131***	0.013
*College enrollment*						
Enrolled in college	−0.321***	0.007	−0.309***	0.006	−0.373***	0.006
*Interaction*						
Birthplace *x* College	−0.425***	0.018	−0.464***	0.017	−0.420***	0.017

*** *p* < 0.001,

** *p* < 0.01,

* *p* < 0.05

**Table 4:** Probit selection equation results for female likelihood of employment.

	Cohort 1 (observed 2006–11)		Cohort 2 (observed 2009–14)		Cohort 3 (observed 2012–17)	
Variables	*β*	SE	*β*	SE	*β*	SE
*Highest education level*						
Associate degree	0.363***	0.012	0.433***	0.011	0.435***	0.010
Bachelor’s degree	0.481***	0.008	0.585***	0.007	0.579***	0.007
Graduate degree	0.592***	0.011	0.693***	0.010	0.694***	0.010
*Observation year*						
Year 2	0.047***	0.009	−0.012	0.008	0.116***	0.008
Year 3	0.070***	0.010	−0.001	0.009	0.171***	0.009
Year 4	−0.032***	0.010	0.019**	0.009	0.196***	0.009
Year 5	−0.128***	0.010	0.024***	0.009	0.239***	0.009
Year 6	−0.174***	0.010	0.011	0.010	0.179***	0.010
*Gave birth*						
Yes, previous 12 mo.	−0.495***	0.009	−0.470***	0.009	−0.481***	0.009
*Birthplace*						
Outside the U.S.	−0.600***	0.010	−0.627***	0.010	−0.654***	0.010
*College enrollment*						
Enrolled in college	−0.109***	0.007	−0.143***	0.006	−0.210***	0.006
*Interaction*						
Birthplace x College	0.001	0.016	0.081***	0.015	0.128***	0.015

*** *p* < 0.001,

** *p* < 0.01,

* *p* < 0.05

**Table 5:** OLS outcome equation results for male estimated mean annual log wage.

Variables	Cohort 1 (2006)		Cohort 2 (2009)		Cohort 3 (2012)	
	*β*	SE	*β*	SE	*β*	SE
*Highest education level* ^†^						
Associate degree	0.519	—	0.510	—	0.453	—
Bachelor’s degree	0.786	—	0.804	—	0.761	—
Graduate degree	1.047	—	1.055	—	1.019	—
*Race/ethnicity*						
Asian	0.048***	0.009	0.066***	0.009	0.041***	0.008
Black	−0.140***	0.007	−0.140***	0.007	−0.152***	0.007
Hispanic/Latino	0.073***	0.007	0.079***	0.007	0.050***	0.006
*Work experience*						
Years of experience	0.182***	0.006	0.076***	0.006	0.086***	0.005
Years squared	−0.012***	0.001	−0.002	0.001	0.000	0.001
*Work intensity*						
Working part-time	−1.024***	0.005	−1.047***	0.005	−1.053***	0.004
*Job prestige score*						
Greater than median	0.237***	0.005	0.217***	0.005	0.223***	0.005
*[Intercept]*	9.806***	0.013	10.015***	0.015	9.968***	0.014
**Goodness-of-fit**						
Inv. Mills ratio (SE)	−1.850*** (0.032)		−1.732*** (0.029)		−1.631*** (0.025)	
*sigma*	1.380		1.384		1.321	
*rho*	−1.340		−1.252		−1.235	
Multiple R^2^	0.530		0.545		0.571	
RMSE	0.820		0.845		0.819	
Obs. (censored)	229,522 (30,717)		250,959 (44,821)		265,166 (47,381)	

*** *p* < 0.001,

** *p* < 0.01,

* *p* < 0.05

^†^ Betas are corrected for the conditional effect of education attainment level on selection into employment.

**Table 6:** OLS outcome equation results for female estimated mean annual log wage.

	Cohort 1 (2006)		Cohort 2 (2009)		Cohort 3 (2012)	
Variables	*β*	SE	*β*	SE	*β*	SE
*Highest education level* ^†^						
Associate degree	0.547	—	0.523	—	0.462	—
Bachelor’s degree	0.919	—	0.919	—	0.875	—
Graduate degree	1.233	—	1.242	—	1.198	—
*Race/ethnicity*						
Asian	0.010	0.009	0.018**	0.009	0.039***	0.008
Black	−0.085***	0.007	−0.050***	0.006	−0.087***	0.006
Hispanic/Latino	0.057***	0.007	0.074***	0.007	0.050***	0.006
*Work experience*						
Years of experience	0.188***	0.004	0.107***	0.004	0.123***	0.004
Years squared	−0.015***	0.001	−0.004***	0.001	−0.004***	0.001
*Work intensity*						
Working part-time	−1.020***	0.005	−1.031***	0.005	−1.031***	0.005
*Job prestige score*						
Greater than median	0.191***	0.005	0.171***	0.005	0.169***	0.005
*[Intercept]*	9.317***	0.010	9.475***	0.011	9.517***	0.012
**Goodness-of-fit**						
Inv. Mills ratio (SE)	−0.547*** (0.024)		−0.651*** (0.023)		−0.816*** (0.022)	
*Sigma*	0.964		1.001		1.045	
*rho*	−0.568		−0.650		−0.781	
Multiple R^2^	0.526		0.535		0.558	
RMSE	0.822		0.837		0.836	
Obs. (censored)	255,921 (41,463)		275,688 (54,317)		285,845 (56,731)	

*** *p* < 0.001,

** *p* < 0.01,

* *p* < 0.05

^†^ Betas are corrected for the conditional effect of education attainment level on selection into employment.

#### Model fit

Goodness-of-fit statistics at the bottom of Tables 5–6 show that selection bias was present in the model data; the inverse Mills ratio (IMR), which is given by *sigma* * *rho*, where *sigma* is the standard error of the residuals in the probit equation and *rho* estimates the correlation between the residuals of the probit and OLS equations, was highly significant. IMR was also negative, informing us that correcting for censored individuals (i.e., controlling for selectivity into employment) reduced the mean size of the annual wage. This is consistent with the notion of the *reservation wage*—the lowest wage a person will accept for a particular job—and its role in a person’s decision to work during a recession. We can think of censored individuals in our data as potential workers whose reservation wages had not been met and thus chose not to enter the labor force. This does not of course mean that every censored young adult in our data *preferred* not to work; during periods of mass job loss and weak hiring, the decision not to work is often made reluctantly. We might instead think of the rapid loss of jobs after 2008 as equivalent to the rapid production of (theoretical) jobs whose wage rate is $0 and the increase in “voluntary” joblessness as the expected outcome when workers are unwilling or unable to lower their reservation wage to $0. Absent a recession, many of these censored individuals would have found jobs that met or exceeded their reservation wage, thus making themselves observable to us.

The negatively-signed IMRs indicate that the contribution of those workers’ wages would have pulled the overall mean wage higher. In Table 5, we can see that the proportion of nonworkers (censored observations) by cohort rose sharply after 2008—the C1 male cohort had fewer censored individuals (30,717 out of 229,522, or 13.4%) than the C2 or C3 cohorts, both of which were 17.9% censored. The proportion of employed males fell after recession onset, and we may infer that this was partly because displaced workers could not find replacement jobs that met their (nonzero) reservation wage. Note that while females also experienced a recession-timed increase in unemployment (Table 6), it was a smaller climb from a higher setpoint: C1 females were 16.2% censored, compared to C2 and C3 females censored at 19.7 and 19.8%, respectively. Female workers appear to have been slightly more likely than males to remain out of the labor force and/or find jobs that met their reservation wage.

Two other goodness-of-fit measures shown in Tables 5–6 apply specifically to the OLS equation. Multiple R^2^ ranged from 0.526 to 0.571, indicating that over half of the variance in the log wages in each cohort was explained by the bias-corrected OLS model. Similarly, the root mean square error (RMSE) of residuals for each cohort ranged between 0.82 to 0.845, indicating mean variance of less than 1 log unit. All these measures were smaller than their equivalents generated by uncorrected OLS regression [not shown], indicating that the Heckit model was a better fit to the data.

#### Probit results for selection into employment

Probit equation coefficients represent the values that maximize the likelihood function for producing the IMRs used to correct for selection bias in the outcome equation. Since these IMRs represent a latent (unobservable) variable, there is no direct interpretation for the coefficients that produced them. We can, however, evaluate them more generally, in terms of whether they were abstractly associated with a greater or lesser likelihood that an individual would be employed.

Certain probit terms had consistently negative effects across cohorts and were representative of conditions little changed by the 2008–09 recession. For males (Table 3), these were birthplace, current enrollment in college, and the interaction between the two effects. For females (Table 4), these were birthplace, current enrollment in college, and having given birth in the previous year. The negative effects on employment likelihood for male college students born outside the U.S., while significant and substantial, would not be unexpected if many of those students were admitted to the U.S. on F-1 student visas that largely disallow off-campus employment [87]; a condition that would be entirely exogenous to recession conditions. For females, however, college enrollment had a substantively lesser negative impact on employment likelihood than having given birth or being born outside the U.S. By contrast, level of education was a consistently positive effect at the cohort level for males and females. Each higher level of education (relative to high school diploma or GED) was associated with a significantly and substantially greater likelihood of selecting into employment. The strength of this effect was generally larger for males than females and smaller for C1 than C2 or C3.

Unlike with most other variables, there was heterogeneity across cohorts in the period effect, as shown by coefficients for observation year. This effect revealed a recession-induced drag on employment that lasted several years and affected females more than males. Negative coefficients for C1 in the latter half of observation (years 5–6 for males and years 4–6 for females) reflected labor market weakness after 2008. As the only significant negative coefficients to appear in our probit results, they affirm the descriptive evidence in Figs 3A and 4A that timing of labor market entry had a distinct effect on employment. Relative to 2006, individuals in C1 were less likely to be employed in a recession or post-recession year, but for young adults in C2, entering at the bottom of the recession meant that subsequent years’ effects were relative to this nadir—there was nowhere to go but up, even if that climb was slow or delayed. Males in this cohort did not experience a substantive positive period effect on employment until year 4, while females did not experience any before being right-censored. This is consistent with the elevated weakness in hiring that characterized the first few years of the economic recovery [88–89]. By contrast, individuals in C3 showed period effects that were larger and grew more rapidly over time, representing the kind of steady year-over-year improvement seen in a strengthening economy.

#### OLS results for mean annual log wage

In a Heckit model, we interpret the coefficients from the outcome equation as we would for any ordinary OLS regression model (with one exception discussed below)—and because of the corrective effect of the IMR-derived regressor, they will be less biased than the coefficients generated from ordinary OLS models. Table 5 presents the coefficients and standard errors for male cohorts, and Table 6 contains the same for female cohorts. Most of these coefficients may be directly interpreted as percent changes to the intercept value (the mean annual log wage for the reference group) when a binary variable is equal to 1 or, in the case of continuous variables, for each additional year of experience [26]. Note that this guideline does not hold well for coefficients more extreme than about +/−0.2 (e.g., education attainment level, working part-time) and that these larger effects are discussed below after the appropriate log-linear transformation.

As seen earlier in the probit equation, regressor coefficients in the wage equation tended to be consistent in size, sign, and significance across cohorts. Compared to non-Hispanic white males, and all else equal, Asian and Hispanic males’ mean log wages were 4.1 to 7.9% higher, depending on cohort, while black males’ log wages were approximately 14 to 15% lower (Table 5). Female cohorts showed a similar pattern (Table 6), although the penalty for black females was substantially lower. Working part-time was especially unfavorable; a coefficient of −1.02 or −1.03 translates into earning 64% less than the reference group (i.e., full-time workers) regardless of cohort or sex. Working a job with an occupational prestige score in the upper half of the distribution increased the log wage by up to one-fifth for female workers and around one-quarter for male workers. These estimated effects did not much vary with recession timing. Work experience, on the other hand, was strongly heterogeneous, with C1 males and females experiencing distinct recession-based patterns not found among their C2 or C3 peers.

In Table 5, we find that the mean C1 male log wage grew by 20% with each additional year of experience (found by taking the natural log of 1 + [coefficient = 0.182]) holding all else constant. (Note that we count the initial observation year as 0 years of experience.) However, as these workers traversed the second half of their observation period, the wage premium for additional years of experience was increasingly offset by the period effect conveyed in the years-squared term. In year 3 (2008), the coefficient of the linear effect for having accumulated two years of experience (0.182 * 2 = 0.364) was offset 13.2% by the nonlinear effect (−0.012 * 4 = −0.048), bringing the adjusted coefficient to 0.316. This equates to a 2008 mean wage that was 37.2% larger than the 2006 mean wage after the log conversion. In year 4, the coefficient for having three years of experience (0.182 * 3 = 0.546) was offset 19.7% by the nonlinear effect (−0.012 * 9 = −0.108) to 0.438, equivalent to net growth of 55% of the 2006 wage after the log conversion. In 2010, after four years of experience, the net gain to the annual wage was equal to 70.9% of the 2006 wage; in 2011, it was equal to 84%.

These male work experience coefficients are readily interpreted as recession effects: males who entered the labor market in 2006 had a few years to accumulate work experience in a strong economy before the recession occurred, with each additional year earning a high wage premium. The size of the linear effect was substantively smaller for C2 and C3 males, but nonetheless continued to be positive and significant. What did *not* persist for C2 and C3 males was the penalty for accumulated experience (shown in the years-squared term), which can be attributed to the recession’s trough occurring either at the moment of labor market entry (for C2) or three years prior (for C3). When we began observing these groups of workers, the worst of the recession had already passed, if only barely; both cohorts were thus moving *away* from the recession instead of toward it. By comparison, work experience effects for female cohorts resembled those for males, but in a muted fashion that showed limited heterogeneity at the cohort level. Female cohorts’ mean log wages gained more with each additional year of work relative to male cohorts and the disparity between female C1 and C2/C3 linear effects was narrower. And while the negative nonlinear effect on C2 and C3 mean wages did not disappear for females, it was greatly reduced relative to C1.

#### Interpretation of the endogenous education attainment variable

The final OLS equation variable to discuss—education attainment—also appears in the probit equation, making it the lone endogenous regressor in our Heckit model. As a result, this variable’s effect on log wage must be adjusted to account for the fact that its effect on selecting into a wage-earning state (employment) had previously been estimated [90–91]. Tables 5–6 show the marginal effects for highest education level after being adjusted downward using the approach by Sigelman & Zeng [91].

Even after correction, it is clear that education was a major wage determinant across cohorts and survey years. Corrected effects show that female workers (Table 6) experienced relatively greater returns to schooling than male workers (Table 5): relative to an HSD/GED, female workers with bachelor’s degrees enjoyed a wage premium equal to 140–150% of the HSD/GED group’s mean wage (after log-linear transformation), compared to only 114–123% for males with bachelor’s degrees. While a graduate degree raised male workers’ mean log wage to 177–187% of the value of an HSD/GED, females’ mean log wage grew more than 230% for the same credential. This was a universal outcome for both sexes, regardless of recession effects.

## Discussion

In this study, we examined how the timing of the Great Recession affected young adults (on a traditional-student pathway) entering the labor force and accumulating work experience in the years before, during, and/or after the recession. We gave particular attention to analyzing labor outcomes on an education gradient to see what kind of recession protection was afforded to individuals who invested in higher education. Could we identify differences in longitudinal trends based on when cohorts interacted with the recession or the recovery? Would those differences vary across the education gradient—and if they did, what would that signify?

We generally expected to see two patterns: (1) labor market outcomes would favor workers with higher degrees regardless of when one entered the market or encountered the recession; and (2) recession effects would be seen across the entire gradient, but in ways that reflected each cohort’s unique exposure to the recession. Our findings confirmed the first pattern but only partially substantiated the second pattern, signifying that even the most severe economic downturn in generations could not repeal the structural nature of young adults’ early career outcomes, despite appearing capable of imposing its own long-term effects. We illustrate this point below in three takeaways.

### Takeaway 1: The recession’s most important effects on employment were lasting ones

The 2008–09 recession had immediate effects on the two cohorts that directly encountered the recession. Most pre-recession (C1) groups lost momentum in the recession, resulting in lower employment rates at year 6 than their peers in later cohorts (an outcome we did not expect) and all recession (C2) groups had lower employment at the start of observation than their peers in C1 (an outcome we did expect). These findings corroborate previous evidence that young workers were heavily displaced by the recession (e.g., [92–93]) and should not be discounted. But the recession’s larger legacy on employment may rest with how certain groups’ trajectories appeared permanently altered after 2008.

This “before/after” effect is seen in the steeper decline in employment rates for all C1 female groups during the latter half of observation relative to later cohorts (Fig 3A) and in the flat employment trajectories of C1 males with less than a bachelor’s degree relative to later cohorts (Fig 4A). It might also be shown by the fact that almost no C1 group, male or female, was able to recover enough momentum by 2011 to attain the employment rates of C2 or C3 at the end of observation, although this may not be a fair comparison given that C1 was unique in experiencing the recession toward the end of their observation period. More worrisome might be the recession’s lasting effect on the employment of young adults with bachelor’s degrees. C1 female employment in this group fell faster after 2008 than for C1 females with associate or graduate degrees. Females with bachelor’s degrees were the only group in C2 to show decline during observation, which strikes us as curious considering that they entered the labor market in its weakest state. For males with bachelor’s degrees, the recession marked the start of a widening employment gap with graduate degree-holders that began in C1 but continued across C2 and C3.

These findings of employment weakness potentially argue for a small yet meaningful devaluation of four-year degrees in the recession and post-recession labor markets. While far from conclusive, they are of a piece with the larger education coefficients seen in the probit selection equations for C2 and C3 relative to C1 (Tables 3–4) that suggest a persistently greater tendency after 2008 for young adults with more than a high school diploma to choose employment on lesser terms than they might prefer (e.g., part-time instead of full-time, lower wage/salary) [94].

### Takeaway 2: Labor outcomes were influenced less by the recession than by education attainment or sex

Lasting or not, it must be acknowledged that the 2008–09 recession’s effects on employment and wages paled in comparison to the distinct and sometimes large disparities in outcomes by education or sex. Young adults with high school diplomas or GEDs were consistently the least employed and lowest earning groups by a wide margin and the recession produced no meaningful change to that relationship. Nor did the recession greatly disturb the high employment or wages of graduate degree-holders or modify the shape of the education gradient across cohorts (notwithstanding the slight wage-damping effect on C2). Between 2006 and 2012, young adults with a given level of education entered observation at about the same starting median wage and left observation five years later with roughly the same higher median wage (Figs 5–6). Such consistency over time was paralleled by the nearly unwavering (and enormous) dispersion in median wages across levels of the education gradient, which was far greater than any difference in wages for a given education level between two cohorts. Equally consistent were the weaker outcomes for females relative to males at a given level of education on almost every employment or wage metric. Even the closure of the gender wage gap for PT workers (Figs 5–6) is diminished by PT workers’ much smaller mean annual wages and female overrepresentation.

The structural nature of education level and sex in shaping labor outcomes has been long established and our findings illustrate how little the recession appears to have changed this. At the broad level of our analysis, the sex-specific education—labor outcome gradients that existed before 2008 continued through the recession intact. This may seem perplexing and frustrating from a gender equity perspective, particularly since females in our analytic sample earned postsecondary credentials at higher rates and in greater numbers than males. The dearth of contextual variables in our data means we cannot prove or disprove that female workers were systematically disadvantaged before, during, or after the recession, but our results are consistent with the literature (e.g., [95–98]) and with other sex-specific discrepancies in our data, including the contrary motion of employment rates over time for male and female graduate degree-holders and the unique susceptibility of female workers with bachelor’s degrees to exit employment. These discrepancies clearly warrant further investigation.

### Takeaway 3: The recession may have precipitated changes that point to the emergence of a “new normal” in the labor market

Several discontinuities in the data at the cohort level show that the recession wrought small yet persistent changes after 2008, offering clues about how young adults adapted to a post-recession labor market. Because many of these discontinuities were subtle, their repercussions may not become readily visible for years, but in the aggregate, they suggest that the recession marked the beginning of a “new normal” for young adults entering the market. For example, the diminishing wage return on years of experience for C2 and C3 workers (Tables 5–6), and on job prestige for C2 and C3 females (Table 6), may be indicative of employers broadly exploiting recessionary conditions to permanently increase their wage setting ability [99]. If validated, this could have implications for workers’ long-term earning power and household wealth. The sharp rise in the proportion of NEET among C1 males appeared to reset the default NEET proportion for C2 and C3 males as well (Fig 4B), suggesting that the recession had evolved the economy in ways that systematically removed even more young males from employment or postsecondary schooling. While it is not possible from our data to know which males were affected or whether they became NEET intentionally, the persistence of a newly higher proportion of male NEET suggests a structural shift.

Perhaps the most significant example of a recession-induced structural shift, however, is in the growth pattern in the proportion of college students who did not work. The large gain in the student only proportion at first observation between 2006 and 2009 was followed by another in 2012 (Figs 3B and 4B). The rise in the student only proportion in 2009 is consistent with the “warehousing” of young adults in college during the 2008–09 recession [20, 100] and explains the sudden drop in the proportion of high school graduates in employment after 2008. But the further climb in the student only proportion in 2012, when the economic recovery was gathering pace, speaks to something else. Since the total proportion of college-goers (student only and student/worker) remained relatively consistent across cohorts, it is unclear if the greater proportion of student only represents a benign or worrying change. For example, it may characterize a greater interest among high school graduates from the classes of 2011 and 2012 to focus on college before turning their attention to a highly competitive labor market (perhaps based on a lingering unease about employment prospects without a higher degree after having witnessed several years of anemic economic recovery). This could be benign if higher education—already well-established as a priority for many young adults in the U.S. [101]—was simply prioritized by an even larger number of high school graduates. On the other hand, it could indicate a mounting financial burden if some of these students borrowed more to pay for college than they would have liked because of meager post-recession job opportunities for workers with only an HSD/GED. If the return on investment for bachelor’s degrees (in the aggregate) is faltering, it would raise the stakes for student borrowers.

### Limitations and next steps

We recognize that our findings and takeaways are based on aggregate data and should be interpreted with care. Our creation of a pseudo-panel data set and use of the Heckit model were intended to improve the longitudinal analysis of cross-sectional data and reduce confounding from latent selection bias, but this approach (as with almost any empirical method) has limitations. For example, we caution against interpreting the results of trajectories of HSD/GED workers in the same way as higher-educated groups. This is because around one-fifth of each male cohort (Fig 2A) and one-quarter of each female cohort (Fig 1A) was lost to attrition during the observation period and most of that attrition was among high school graduates earning postsecondary degrees. Thus, labor outcomes for HSD/GED groups in year 6 were more representative of the typical high school graduate with no intentions for college than they were in years 1–3, as the composition of HSD/GED groups in years 1–3 necessarily included individuals who later earned postsecondary credentials and would leave our cohort. While under observation, college-going high school graduates may have helped lower the HSD/GED employment rate (if they were student only) or drag down the median annual wage (if they were student/worker and were working simply to help cover living expenses). This could confound the reliability of estimated outcomes given the majority share of each cohort made up by the HSD/GED group. (Note that this concern is less applicable to individuals with postsecondary credentials because of their substantially lower attrition rates).

Beyond this, our longitudinal conclusions are limited in the usual ways for cross-sectional data. Developing our findings using context-rich panel data would be a natural next step that also opens the door to the causal analysis of patterns and associations revealed in this study. Other important directions include accounting for the wide variability implied by conditions such as “employment” or “education,” and addressing the endogeneity of labor market timing. Regarding the former, the lack of a large deviation in outcomes across cohorts at the high end of the education gradient implies a protective education effect, but offers no insights into important aspects of labor, including job quality and underemployment, that could have broadly changed after recession onset but were not measured in our data. On the latter, our cohort design limits generalizability to young adults on a traditional-student trajectory, which means our findings cannot necessarily speak to individuals who deviated substantially from this trajectory. It will be valuable to analyze in future studies what proportion of young adults delayed their graduation from college to avoid a direct encounter with the recession and what their outcomes were as a result.

Limitations notwithstanding, as a first look at the labor outcomes and trajectories of young workers around the time of the 2008–09 recession, this study offers new evidence for the differentiation of early-career trajectories by recession proximity and lays out several promising research directions. Timing mattered, but so did educational attainment and much else too, and it remains to be seen what the true legacy of the Great Recession will be for young adults who started their careers in the recession’s wake.
